# Chronic Wounds and Their Therapy with Alginate-Based Dressings

**DOI:** 10.3390/jpm12091356

**Published:** 2022-08-24

**Authors:** Raimund Winter

**Affiliations:** Division of Plastic, Aesthetic and Reconstructive Surgery, Department of Surgery, Medical University of Graz, Auenbruggerplatz 5, 8036 Graz, Austria; r.winter@medunigraz.at

This invited editorial paper is intended to provide a brief overview of the use of alginate-based wound dressings in the treatment of chronic wounds, focusing particularly on the review by Andreea Barbu et al. entitled “Current Trends in Advanced Alginate-Based Wound Dressings for Chronic Wounds” [1]. This review has recently achieved the rank of a highly cited paper.

Chronic wounds are a costly burden to the healthcare system. The annual cost of treating chronic wounds in the United States is estimated to be USD 25 billion, with more than 2.5 million patients receiving treatment for chronic wounds each year [2]. 

The high cost of chronic wounds is due to a variety of factors, including the need for frequent dressing changes, the use of expensive wound dressings, and the need for extended hospital stays for some patients. 

Chronic wounds are a challenge for patients and their families. These wounds often lead to a decrease in quality of life for patients, as they may experience pain, disfigurement, and a loss of mobility [3].

Current standards of care include debridement, infection control, and topical wound dressings [4]. One of the most important and most frequently used types of wound dressing is alginate-based wound dressings. First described in 1928, alginates continue to be used in the modern treatment of chronic wounds due to their versatility [5].

Alginate-based wound dressings are derived from brown algae and are composed of long chains of guluronic and mannuronic acid [6]. These dressings are able to absorb large amounts of exudate and maintain a moist wound environment, which is essential for healing [7,8]. Alginate-based wound dressings can be left in place for up to 7 days, are non-adherent, do not cause skin irritation, are available in a variety of sizes, and are relatively inexpensive [9].

In addition, alginate-based dressings promote the formation of new granulation tissue, provide a scaffold for cell migration, stimulate fibroblast proliferation and collagen synthesis, and thereby promote wound healing [10,11,12,13].

Several studies have evaluated the use of alginate-based dressings in the treatment of chronic wounds and have shown promising results [14,15].

Alginate-based dressings are a safe and effective option for the management of chronic wounds.

The recent review “Current Trends in Advanced Alginate-Based Wound Dressings for Chronic Wounds“ by Barbu et al. brilliantly summarizes the versatility and advantages of alginate-based wound dressings [1].

The authors describe the physical properties of alginates and how they are chemically structured. They explain in detail in which processing forms alginate-based dressings occur, for example, as beads and microcapsules, as nanofibers, or as hydrogels.

Depending on their chemical structure, hydrogels can be soft and flexible or rigid with strong mechanical resistance [16]. In a multilayer structure, they can also act as a carrier medium for cells and thus promote tissue regeneration [17].

The authors describe how alginates promote wound healing and the possibilities to further improve their antimicrobial and antifungal properties, for example, by treating alginates with silver ions or zinc oxide nanoparticles [11].

In conclusion, chronic wounds are a widespread and costly problem. Alginate-based dressings are easy to apply and effective in the treatment of chronic wounds.

Barbu et al. provide a perfect overview of the different possibilities of using alginate-based dressings.
